# Supplementary material for: A Likelihood Approach for Real-Time Calibration of Stochastic Compartmental Epidemic Models
Source: PLoS Comput Biol. 2017 Jan 17;13(1):e1005257. doi: 10.1371/journal.pcbi.1005257 (PMC5240920; doi:10.1371/journal.pcbi.1005257)
Supplement: S1 File — (TAR.GZ) [file pcbi.1005257.s014.tar.gz › HSPH_Online-SI-Revision/EnKF07/n10-extreme/n10-extreme_EnKF2_table4.pdf]

1	newly 3 weeks spec	135.	60.73	{12., 115.}	84.0667	0.725084	newly 3 weeks cumul	629.	244.47	{73., 345.}	76.6789	0.666603	newly 5 weeks spec	61.	48.64	{1., 76.}	116.426	0.929043	newly 5 weeks cumul	793.	346.78	{89., 623.}	81.3796	0.681381	newly 54 weeks cumul	895.	588.42	{99., 1387.}	99.1799	0.69574
2	newly 3 weeks spec	274.	167.28	{4., 382.}	102.978	0.977849	newly 3 weeks cumul	1010.	1971.39	{112., 6753.}	170.896	0.477776	newly 5 weeks spec	172.	26.91	{0., 54.}	93.4244	1.91609	newly 5 weeks cumul	1394.	2064.07	{112., 7428.}	142.751	0.542387	newly 50 weeks cumul	1905.	2084.23	{112., 7428.}	120.929	0.620047
3	newly 3 weeks spec	87.	28.82	{0., 27.}	107.563	1.82253	newly 3 weeks cumul	473.	490.01	{164., 472.}	98.3911	0.374991	newly 5 weeks spec	29.	7.09	{0., 7.}	105.966	2.15416	newly 5 weeks cumul	554.	509.84	{165., 561.}	97.5921	0.426378	newly 56 weeks cumul	592.	521.43	{165., 653.}	97.5895	0.448205
4	newly 3 weeks spec	199.	184.33	{8., 290.}	126.668	0.822629	newly 3 weeks cumul	783.	1176.98	{86., 2596.}	140.943	0.482632	newly 5 weeks spec	95.	52.27	{0., 137.}	107.632	1.33101	newly 5 weeks cumul	1007.	1326.48	{93., 2758.}	133.648	0.535901	newly 50 weeks cumul	1308.	1406.42	{99., 2875.}	121.483	0.606641
5	newly 3 weeks spec	213.	70.82	{0., 120.}	97.8873	1.89621	newly 3 weeks cumul	916.	407.4	{18., 774.}	91.2926	0.887704	newly 5 weeks spec	132.	59.53	{0., 63.}	110.371	1.89811	newly 5 weeks cumul	1203.	534.13	{18., 1006.}	93.8263	0.927643	newly 53 weeks cumul	1516.	896.87	{24., 2697.}	104.114	0.946006
6	newly 3 weeks spec	232.	72.22	{10., 73.}	90.6552	0.931267	newly 3 weeks cumul	932.	275.34	{53., 276.}	84.3283	0.813165	newly 5 weeks spec	93.	51.74	{2., 48.}	110.86	1.00183	newly 5 weeks cumul	1166.	389.04	{69., 369.}	87.3396	0.818714	newly 54 weeks cumul	1415.	608.58	{103., 580.}	96.5272	0.834301
7	newly 3 weeks spec	196.	91.24	{0., 102.}	107.306	1.88788	newly 3 weeks cumul	905.	962.9	{82., 3289.}	112.833	0.570653	newly 5 weeks spec	97.	31.29	{0., 54.}	96.3402	1.78804	newly 5 weeks cumul	1165.	1047.42	{97., 3817.}	106.124	0.61171	newly 55 weeks cumul	1418.	1096.31	{105., 4063.}	99.6664	0.643191
8	newly 3 weeks spec	285.	43.95	{1., 86.}	91.0702	1.38034	newly 3 weeks cumul	1128.	568.35	{52., 1262.}	84.5505	0.725841	newly 5 weeks spec	172.	5.87	{0., 14.}	96.5872	2.28437	newly 5 weeks cumul	1506.	589.42	{53., 1288.}	83.008	0.809781	newly 52 weeks cumul	1911.	593.22	{53., 1291.}	83.2507	0.894655
9	newly 3 weeks spec	224.	52.44	{6., 90.}	77.3125	0.793456	newly 3 weeks cumul	973.	440.5	{168., 657.}	70.3515	0.526092	newly 5 weeks spec	131.	20.44	{0., 41.}	84.3969	1.29238	newly 5 weeks cumul	1278.	491.33	{199., 705.}	72.9992	0.57594	newly 52 weeks cumul	1632.	548.71	{219., 724.}	74.9063	0.624405
10	newly 3 weeks spec	311.	97.11	{8., 157.}	83.3859	0.871924	newly 3 weeks cumul	1154.	1314.79	{220., 1358.}	130.318	0.550404	newly 5 weeks spec	185.	22.04	{0., 53.}	88.0865	1.45463	newly 5 weeks cumul	1603.	1375.45	{244., 1396.}	114.263	0.610381	newly 52 weeks cumul	2165.	1421.24	{255., 1400.}	104.03	0.677464
11	newly 3 weeks spec	280.	100.5	{0., 401.}	86.0143	2.14491	newly 3 weeks cumul	1095.	1860.86	{503., 3157.}	118.687	0.301224	newly 5 weeks spec	155.	54.61	{0., 201.}	88.5097	2.1063	newly 5 weeks cumul	1475.	1985.7	{656., 3241.}	103.591	0.35225	newly 47 weeks cumul	1957.	2202.06	{656., 4500.}	95.5902	0.40726
12	newly 3 weeks spec	241.	97.9	{12., 176.}	77.3195	0.706336	newly 3 weeks cumul	1116.	1362.74	{244., 1126.}	136.111	0.536923	newly 5 weeks spec	113.	24.06	{0., 52.}	80.3363	1.15282	newly 5 weeks cumul	1420.	1430.85	{271., 1241.}	121.46	0.568366	newly 52 weeks cumul	1677.	1485.15	{280., 1440.}	112.729	0.592192
13	newly 3 weeks spec	180.	243.01	{46., 297.}	118.961	0.387903	newly 3 weeks cumul	742.	863.02	{258., 1071.}	84.8005	0.299001	newly 5 weeks spec	114.	130.55	{7., 180.}	123.482	0.588532	newly 5 weeks cumul	985.	1182.63	{350., 1491.}	95.3046	0.329462	newly 48 weeks cumul	1277.	1506.33	{403., 3050.}	98.9561	0.355057
14	newly 3 weeks spec	185.	84.21	{0., 173.}	103.692	1.75492	newly 3 weeks cumul	825.	474.07	{29., 1029.}	93.3952	0.812187	newly 5 weeks spec	104.	59.9	{0., 64.}	120.154	1.85072	newly 5 weeks cumul	1066.	601.05	{37., 1588.}	95.6726	0.842394	newly 53 weeks cumul	1314.	910.97	{51., 2943.}	103.521	0.83068
15	newly 3 weeks spec	149.	97.68	{1., 240.}	115.383	1.13294	newly 3 weeks cumul	603.	749.82	{34., 831.}	152.046	0.726634	newly 5 weeks spec	76.	40.76	{0., 64.}	117.316	1.64708	newly 5 weeks cumul	785.	846.53	{36., 1205.}	141.65	0.788024	newly 50 weeks cumul	962.	970.95	{36., 2435.}	138.843	0.846799
16	newly 3 weeks spec	236.	94.63	{0., 184.}	93.8941	1.7695	newly 3 weeks cumul	954.	777.19	{159., 1729.}	83.7788	0.485387	newly 5 weeks spec	131.	70.8	{0., 106.}	106.473	1.83429	newly 5 weeks cumul	1261.	930.77	{196., 2741.}	84.0849	0.520535	newly 53 weeks cumul	1555.	1283.88	{219., 4157.}	89.0058	0.520434
17	newly 3 weeks spec	218.	59.07	{0., 78.}	96.922	1.89856	newly 3 weeks cumul	974.	837.98	{72., 1395.}	97.306	0.610797	newly 5 weeks spec	107.	22.17	{0., 42.}	92.9439	1.84876	newly 5 weeks cumul	1233.	896.77	{83., 1557.}	88.3998	0.622143	newly 55 weeks cumul	1481.	940.43	{89., 1701.}	84.8947	0.653018
18	newly 3 weeks spec	202.	48.13	{0., 98.}	89.9059	1.88111	newly 3 weeks cumul	887.	503.42	{27., 826.}	103.581	0.878053	newly 5 weeks spec	107.	42.72	{0., 103.}	103.346	1.85646	newly 5 weeks cumul	1132.	590.81	{34., 1138.}	99.8277	0.905785	newly 53 weeks cumul	1431.	900.71	{38., 2936.}	107.516	0.928268
19	newly 3 weeks spec	155.	91.17	{0., 101.}	112.265	1.64885	newly 3 weeks cumul	791.	1092.82	{74., 1296.}	142.399	0.557424	newly 5 weeks spec	62.	52.71	{0., 67.}	131.5	1.51866	newly 5 weeks cumul	957.	1217.59	{84., 1673.}	136.129	0.572595	newly 55 weeks cumul	1067.	1342.29	{94., 2349.}	133.152	0.567269
20	newly 3 weeks spec	277.	56.09	{8., 106.}	82.4513	0.926588	newly 3 weeks cumul	1115.	412.98	{82., 904.}	66.5973	0.60413	newly 5 weeks spec	154.	19.21	{0., 44.}	87.526	1.39152	newly 5 weeks cumul	1485.	464.35	{92., 989.}	71.2747	0.648759	newly 52 weeks cumul	1930.	506.73	{100., 1001.}	75.3093	0.715731
21	newly 3 weeks spec	230.	150.39	{0., 625.}	105.335	1.75386	newly 3 weeks cumul	931.	991.98	{130., 2940.}	95.9291	0.4536	newly 5 weeks spec	137.	59.52	{0., 230.}	91.6496	1.70947	newly 5 weeks cumul	1250.	1146.59	{141., 3544.}	94.1672	0.513792	newly 49 weeks cumul	1622.	1261.24	{141., 3923.}	90.2688	0.573166
22	newly 3 weeks spec	126.	50.62	{6., 93.}	68.8254	0.644085	newly 3 weeks cumul	596.	504.54	{260., 914.}	54.7047	0.269056	newly 5 weeks spec	39.	11.96	{0., 36.}	79.5385	1.24774	newly 5 weeks cumul	702.	541.41	{263., 968.}	54.7279	0.294394	newly 56 weeks cumul	785.	555.32	{263., 1002.}	55.5796	0.320814
23	newly 3 weeks spec	183.	160.73	{9., 220.}	109.503	0.684693	newly 3 weeks cumul	736.	698.65	{45., 912.}	102.346	0.614605	newly 5 weeks spec	84.	90.98	{5., 118.}	139.81	0.785201	newly 5 weeks cumul	938.	912.61	{64., 1244.}	107.43	0.629718	newly 51 weeks cumul	1140.	1116.02	{80., 1548.}	111.439	0.640176
24	newly 3 weeks spec	218.	107.16	{0., 305.}	95.6239	1.69082	newly 3 weeks cumul	857.	830.53	{55., 2028.}	82.7713	0.501825	newly 5 weeks spec	150.	38.05	{0., 100.}	87.02	1.74619	newly 5 weeks cumul	1186.	931.1	{63., 2288.}	82.7454	0.570383	newly 49 weeks cumul	1579.	1014.79	{65., 2702.}	81.4408	0.637682
25	newly 3 weeks spec	123.	127.86	{4., 153.}	113.789	0.663221	newly 3 weeks cumul	613.	739.03	{131., 987.}	99.5775	0.382714	newly 5 weeks spec	53.	37.84	{0., 68.}	98.6038	1.14495	newly 5 weeks cumul	719.	839.57	{141., 1025.}	97.6147	0.3884	newly 52 weeks cumul	805.	1004.82	{141., 1439.}	106.788	0.40761
26	newly 3 weeks spec	82.	36.82	{0., 55.}	109.829	1.66231	newly 3 weeks cumul	370.	788.03	{43., 709.}	217.138	0.613972	newly 5 weeks spec	10.	17.76	{0., 14.}	215.8	1.20432	newly 5 weeks cumul	409.	832.6	{51., 828.}	208.215	0.595947	newly 55 weeks cumul	439.	866.47	{56., 828.}	203.073	0.592235
27	newly 3 weeks spec	253.	96.53	{0., 113.}	97.9723	1.81136	newly 3 weeks cumul	1055.	712.78	{179., 1740.}	84.	0.532063	newly 5 weeks spec	136.	74.54	{0., 67.}	114.485	1.81234	newly 5 weeks cumul	1391.	872.68	{198., 3207.}	87.0554	0.575379	newly 53 weeks cumul	1730.	1184.49	{219., 3390.}	93.9902	0.595115
28	newly 3 weeks spec	143.	48.37	{0., 44.}	114.748	2.18638	newly 3 weeks cumul	714.	493.08	{155., 670.}	94.9384	0.55216	newly 5 weeks spec	59.	19.96	{0., 6.}	119.322	2.49683	newly 5 weeks cumul	874.	543.56	{155., 692.}	97.3776	0.624485	newly 56 weeks cumul	975.	580.72	{155., 692.}	99.1836	0.664589
29	newly 3 weeks spec	161.	300.46	{1., 1404.}	228.919	1.23308	newly 3 weeks cumul	747.	1458.07	{34., 6358.}	196.719	0.675657	newly 5 weeks spec	83.	40.52	{0., 105.}	118.771	1.98467	newly 5 weeks cumul	974.	1627.89	{34., 8202.}	180.048	0.74084	newly 51 weeks cumul	1110.	1642.07	{34., 8284.}	166.176	0.773529
30	newly 3 weeks spec	187.	108.49	{0., 211.}	113.684	1.83487	newly 3 weeks cumul	802.	1226.56	{90., 3738.}	158.863	0.584618	newly 5 weeks spec	78.	25.43	{0., 59.}	97.859	1.88749	newly 5 weeks cumul	1008.	1303.37	{91., 3906.}	143.775	0.63859	newly 51 weeks cumul	1234.	1341.13	{91., 3942.}	131.706	0.697973
31	newly 3 weeks spec	252.	113.95	{9., 112.}	104.742	0.987671	newly 3 weeks cumul	969.	357.8	{37., 384.}	90.7967	0.82753	newly 5 weeks spec	111.	77.54	{2., 93.}	124.414	1.06304	newly 5 weeks cumul	1242.	533.74	{54., 581.}	95.9694	0.844362	newly 54 weeks cumul	1508.	783.36	{94., 969.}	103.301	0.859957
32	newly 3 weeks spec	205.	119.95	{34., 248.}	76.8537	0.510986	newly 3 weeks cumul	891.	1017.52	{396., 1999.}	74.8283	0.268535	newly 5 weeks spec	111.	27.71	{1., 77.}	81.3423	1.07921	newly 5 weeks cumul	1156.	1101.11	{425., 2206.}	71.1375	0.309315	newly 46 weeks cumul	1578.	1142.68	{426., 2258.}	68.8188	0.384906
33	newly 3 weeks spec	80.	160.93	{21., 323.}	167.738	0.387896	newly 3 weeks cumul	415.	717.07	{209., 994.}	127.342	0.300816	newly 5 weeks spec	25.	96.81	{1., 304.}	357.8	0.730987	newly 5 weeks cumul	481.	935.18	{276., 1828.}	143.996	0.301585	newly 54 weeks cumul	518.	1507.5	{293., 4961.}	234.598	0.361441
34	newly 3 weeks spec	107.	127.2	{0., 126.}	153.159	1.33	newly 3 weeks cumul	518.	831.29	{28., 3011.}	150.782	0.576368	newly 5 weeks spec	37.	55.43	{0., 51.}	186.622	1.54539	newly 5 weeks cumul	629.	969.7	{29., 3011.}	151.431	0.61724	newly 56 weeks cumul	672.	1079.38	{29., 3027.}	158.723	0.631789
35	newly 3 weeks spec	219.	140.01	{24., 200.}	82.9269	0.540726	newly 3 weeks cumul	914.	752.3	{112., 1142.}	96.6565	0.531043	newly 5 weeks spec	143.	56.7	{11., 90.}	78.7972	0.646467	newly 5 weeks cumul	1246.	894.14	{149., 1331.}	90.3242	0.543921	newly 51 weeks cumul	1672.	1062.9	{172., 1451.}	85.2799	0.559229
36	newly 3 weeks spec	304.	151.59	{0., 645.}	92.4309	1.81453	newly 3 weeks cumul	1147.	983.9	{127., 3193.}	81.0881	0.47214	newly 5 weeks spec	161.	61.7	{0., 240.}	84.4099	1.71716	newly 5 weeks cumul	1520.	1139.86	{148., 3675.}	82.0368	0.53123	newly 49 weeks cumul	2076.	1269.44	{160., 3996.}	78.8054	0.595866
37	newly 3 weeks spec	209.	82.6	{3., 122.}	79.8756	0.82454	newly 3 weeks cumul	875.	729.39	{175., 870.}	89.0434	0.443003	newly 5 weeks spec	117.	23.47	{0., 68.}	79.9402	1.37484	newly 5 weeks cumul	1151.	792.23	{193., 931.}	84.8245	0.494196	newly 52 weeks cumul	1404.	846.42	{202., 1051.}	81.5385	0.530408
38	newly 3 weeks spec	55.	17.08	{0., 34.}	79.2364	1.33425	newly 3 weeks cumul	284.	505.77	{53., 765.}	163.75	0.451503	newly 5 weeks spec	18.	4.8	{0., 16.}	81.2222	1.31062	newly 5 weeks cumul	331.	519.82	{62., 833.}	146.218	0.458266	newly 55 weeks cumul	350.	526.91	{64., 854.}	140.654	0.460258
39	newly 3 weeks spec	107.	90.54	{0., 116.}	119.215	1.38249	newly 3 weeks cumul	564.	851.15	{76., 1075.}	140.651	0.465568	newly 5 weeks spec	42.	60.15	{0., 70.}	179.929	1.45444	newly 5 weeks cumul	669.	988.54	{76., 1713.}	140.625	0.488769	newly 55 weeks cumul	739.	1162.66	{76., 2465.}	150.36	0.510796
40	newly 3 weeks spec	222.	61.44	{7., 121.}	74.5315	0.833864	newly 3 weeks cumul	944.	581.84	{200., 984.}	71.7246	0.486419	newly 5 weeks spec	115.	21.7	{0., 63.}	81.1304	1.40563	newly 5 weeks cumul	1200.	639.27	{200., 990.}	70.5908	0.512198	newly 52 weeks cumul	1524.	701.22	{200., 1069.}	70.5538	0.554002
41	newly 3 weeks spec	212.	177.87	{14., 319.}	109.759	0.704926	newly 3 weeks cumul	904.	683.23	{77., 1140.}	100.222	0.62387	newly 5 weeks spec	89.	128.62	{5., 305.}	164.427	0.752256	newly 5 weeks cumul	1155.	956.6	{92., 1687.}	104.952	0.637016	newly 51 weeks cumul	1447.	1630.35	{115., 7321.}	130.777	0.66458
42	newly 3 weeks spec	235.	142.95	{0., 374.}	101.102	1.8344	newly 3 weeks cumul	936.	786.7	{52., 1855.}	86.3013	0.586684	newly 5 weeks spec	165.	87.55	{0., 294.}	103.	1.91023	newly 5 weeks cumul	1287.	982.55	{66., 2343.}	87.7428	0.644417	newly 46 weeks cumul	1777.	1324.96	{67., 4476.}	91.8582	0.716875
43	newly 3 weeks spec	116.	46.97	{4., 97.}	64.181	0.651511	newly 3 weeks cumul	596.	441.14	{171., 826.}	46.6242	0.283326	newly 5 weeks spec	40.	12.43	{0., 38.}	73.075	1.1406	newly 5 weeks cumul	704.	477.17	{172., 835.}	47.8878	0.309675	newly 56 weeks cumul	757.	491.48	{172., 835.}	48.5443	0.323664
44	newly 3 weeks spec	281.	253.06	{11., 378.}	115.445	0.76344	newly 3 weeks cumul	1071.	986.6	{71., 2301.}	97.5182	0.579715	newly 5 weeks spec	173.	145.56	{2., 170.}	131.156	1.02266	newly 5 weeks cumul	1453.	1325.86	{81., 2719.}	104.372	0.629147	newly 47 weeks cumul	1985.	1687.84	{97., 2777.}	107.497	0.694745
45	newly 3 weeks spec	250.	154.19	{22., 266.}	83.876	0.612824	newly 3 weeks cumul	978.	744.58	{109., 1036.}	89.6442	0.544394	newly 5 weeks spec	142.	78.41	{11., 139.}	89.5282	0.730927	newly 5 weeks cumul	1328.	928.07	{139., 1409.}	86.93	0.568154	newly 51 weeks cumul	1725.	1221.03	{188., 1748.}	89.6736	0.589106
46	newly 3 weeks spec	267.	174.74	{0., 657.}	103.251	1.74751	newly 3 weeks cumul	1078.	1167.89	{125., 3717.}	96.0826	0.490285	newly 5 weeks spec	173.	68.58	{0., 262.}	88.5087	1.75663	newly 5 weeks cumul	1493.	1347.29	{148., 4462.}	92.649	0.55594	newly 49 weeks cumul	2102.	1472.32	{155., 4800.}	86.1846	0.635255
47	newly 3 weeks spec	255.	97.63	{0., 222.}	99.8392	1.89572	newly 3 weeks cumul	1074.	1290.41	{42., 3817.}	137.375	0.722751	newly 5 weeks spec	129.	19.72	{0., 55.}	90.9457	2.06916	newly 5 weeks cumul	1366.	1352.27	{49., 3834.}	123.137	0.768675	newly 51 weeks cumul	1768.	1416.63	{49., 3869.}	108.927	0.820877
48	newly 3 weeks spec	231.	61.71	{12., 88.}	85.4156	0.937283	newly 3 weeks cumul	980.	236.39	{60., 310.}	81.048	0.860152	newly 5 weeks spec	109.	47.17	{2., 63.}	100.046	1.01314	newly 5 weeks cumul	1224.	337.31	{75., 445.}	82.6691	0.867112	newly 54 weeks cumul	1459.	570.09	{96., 731.}	92.1775	0.88244
49	newly 3 weeks spec	157.	119.54	{0., 83.}	133.631	1.66422	newly 3 weeks cumul	774.	1237.08	{97., 2853.}	163.16	0.573593	newly 5 weeks spec	60.	48.47	{0., 53.}	133.083	1.55031	newly 5 weeks cumul	908.	1365.33	{132., 3928.}	157.798	0.580469	newly 55 weeks cumul	1013.	1451.42	{137., 4304.}	151.329	0.58033
50	newly 3 weeks spec	221.	115.74	{0., 244.}	99.629	1.72808	newly 3 weeks cumul	1020.	866.06	{191., 2348.}	85.349	0.467598	newly 5 weeks spec	108.	94.	{0., 251.}	129.926	1.72038	newly 5 weeks cumul	1290.	1065.4	{237., 3251.}	89.3922	0.503138	newly 53 weeks cumul	1505.	1479.8	{282., 5525.}	102.258	0.510632
